# Do Pressures to Publish Increase Scientists' Bias? An Empirical Support from US States Data

**DOI:** 10.1371/journal.pone.0010271

**Published:** 2010-04-21

**Authors:** Daniele Fanelli

**Affiliations:** INNOGEN and Institute for the Study of Science, Technology and Innovation (ISSTI), The University of Edinburgh, Edinburgh, United Kingdom; University of East Piedmont, Italy

## Abstract

The growing competition and “publish or perish” culture in academia might conflict with the objectivity and integrity of research, because it forces scientists to produce “publishable” results at all costs. Papers are less likely to be published and to be cited if they report “negative” results (results that fail to support the tested hypothesis). Therefore, if publication pressures increase scientific bias, the frequency of “positive” results in the literature should be higher in the more competitive and “productive” academic environments. This study verified this hypothesis by measuring the frequency of positive results in a large random sample of papers with a corresponding author based in the US. Across all disciplines, papers were more likely to support a tested hypothesis if their corresponding authors were working in states that, according to NSF data, produced more academic papers per capita. The size of this effect increased when controlling for state's per capita R&D expenditure and for study characteristics that previous research showed to correlate with the frequency of positive results, including discipline and methodology. Although the confounding effect of institutions' prestige could not be excluded (researchers in the more productive universities could be the most clever and successful in their experiments), these results support the hypothesis that competitive academic environments increase not only scientists' productivity but also their bias. The same phenomenon might be observed in other countries where academic competition and pressures to publish are high.

## Introduction

The objectivity and integrity of contemporary science faces many threats. A cause of particular concern is the growing competition for research funding and academic positions, which, combined with an increasing use of bibliometric parameters to evaluate careers (e.g. number of publications and the impact factor of the journals they appeared in), pressures scientists into continuously producing “publishable” results [1].

Competition is encouraged in scientifically advanced countries because it increases the efficiency and productivity of researchers [2]. The flip side of the coin, however, is that it might conflict with their objectivity and integrity, because the success of a scientific paper partly depends on its outcome. In many fields of research, papers are more likely to be published [3], [4], [5], [6], to be cited by colleagues [7], [8], [9] and to be accepted by high-profile journals [10] if they report results that are “positive” – term which in this paper will indicate all results that support the experimental hypothesis against an alternative or a “null” hypothesis of no effect, using or not using tests of statistical significance.

Words like “positive”, “significant”, “negative” or “null” are common scientific jargon, but are obviously misleading, because all results are equally relevant to science, as long as they have been produced by sound logic and methods [11], [12]. Yet, literature surveys and meta-analyses have extensively documented an excess of positive and/or statistically significant results in fields and subfields of, for example, biomedicine [13], biology [14], ecology and evolution [15], psychology [16], economics [17], sociology [18].

Many factors contribute to this publication bias against negative results, which is rooted in the psychology and sociology of science. Like all human beings, scientists are confirmation-biased (i.e. tend to select information that supports their hypotheses about the world) [19], [20], [21], and they are far from indifferent to the outcome of their own research: positive results make them happy and negative ones disappointed [22]. This bias is likely to be reinforced by a positive feedback from the scientific community. Since papers reporting positive results attract more interest and are cited more often, journal editors and peer reviewers might tend to favour them, which will further increase the desirability of a positive outcome to researchers, particularly if their careers are evaluated by counting the number of papers listed in their CVs and the impact factor of the journals they are published in.

Confronted with a “negative” result, therefore, a scientist might be tempted to either not spend time publishing it (what is often called the “file-drawer effect”, because negative papers are imagined to lie in scientists' drawers) or to turn it somehow into a positive result. This can be done by re-formulating the hypothesis (sometimes referred to as HARKing: Hypothesizing After the Results are Known [23]), by selecting the results to be published [24], by tweaking data or analyses to “improve” the outcome, or by willingly and consciously falsifying them [25]. Data fabrication and falsification are probably rare, but other questionable research practices might be relatively common [26].

Quantitative studies have repeatedly shown that financial interests can influence the outcome of biomedical research [27], [28] but they appear to have neglected the much more widespread conflict of interest created by scientists' need to publish. Yet, fears that the professionalization of research might compromise its objectivity and integrity had been expressed already in the 19^th^ century [29]. Since then, the competitiveness and precariousness of scientific careers have increased [30], and evidence that this might encourage misconduct has accumulated. Scientists in focus groups suggested that the need to compete in academia is a threat to scientific integrity [1], and those guilty of scientific misconduct often invoke excessive pressures to produce as a partial justification for their actions [31]. Surveys suggest that competitive research environments decrease the likelihood to follow scientific ideals [32] and increase the likelihood to witness scientific misconduct [33] (but see [34]). However, no direct, quantitative study has verified the connection between pressures to publish and bias in the scientific literature, so the existence and gravity of the problem are still a matter of speculation and debate [35].

To verify this hypothesis, this study analysed a random sample of papers published between 2000 and 2007 that had a corresponding author based in the US. These papers, published in all disciplines, declared to have tested a hypothesis, and it was determined whether they concluded to have found a “positive” (full or partial) or a “negative” support for the tested hypothesis. Using data compiled by the National Science Foundation, the proportion of “positive” results was then regressed against a sheer measure of academic productivity: the number of articles published per-capita (i.e. per doctorate holder in academia) in each US state, controlling for the effects of per-capita research expenditure. NSF data provides an accurate proxy of a state's academic productivity, because it controls for multiple authorship by counting papers fractionally. Since the probability for a paper to report a positive result depends significantly on its methodology, on whether it tests one or more hypotheses, on the discipline it belongs to and particularly on whether the discipline is pure or applied [36], these confounding effects were controlled for in the regression models.

## Results

**Figure 1:**
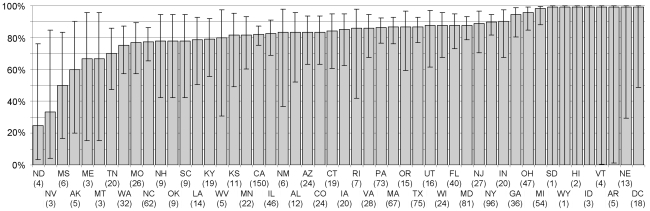
Percentage of positive results by US state. Percentage and 95% logit-derived confidence interval of papers published between 2000 and 2007 that supported a tested hypothesis, classified by the corresponding author's US state (sample size for each state is in parentheses). States are indicated by their official USPS abbreviations: AL-Alabama, AK-Alaska, AZ-Arizona, AR-Arkansas, CA-California, CO-Colorado, CT-Connecticut, DC-District of Columbia, FL-Florida, GA-Georgia, HI-Hawaii, ID-Idaho, IL-Illinois, IN-Indiana, IA-Iowa, KS-Kansas, KY-Kentucky, LA-Louisiana, ME-Maine, MD-Maryland, MA-Massachusetts, MI-Michigan, MN-Minnesota, MS-Mississippi, MO-Missouri, MT-Montana, NE-Nebraska, NV-Nevada, NH-New Hampshire, NJ-New Jersey, NM-New Mexico, NY-New York, NC-North Carolina, ND-North Dakota, OH-Ohio, OK-Oklahoma, OR-Oregon, PA-Pennsylvania, RI-Rhode Island, SC-South Carolina, SD-South Dakota, TN-Tennessee, TX-Texas, UT-Utah, VT-Vermont, VA-Virginia, WA-Washington, WV-West Virginia, WI-Wisconsin, WY-Wyoming. All US states were represented in the sample except Delaware.

A total of 1316 papers were included in the analysis. All US states and the federal district were represented in the sample, except Delaware. The number of papers per state varied between 1 and 150 (mean: 26.32±4.16SE), and the percentage of positive results between 25% and 100% (mean: 82.38±15.15STDV, Figure 1). The number of papers from each state in the sample was almost perfectly correlated with the total number of papers that each state had published in 2003 according to NSF (Pearson's r = 0.968, N = 50, P<0.001), as well as any other year for which data was available (i.e. 1997, 2001 and 2005, r≥0.963 and p<0.001 in all cases). This shows the sample to be highly representative of academic publication patterns in the US.

**Figure 2:**
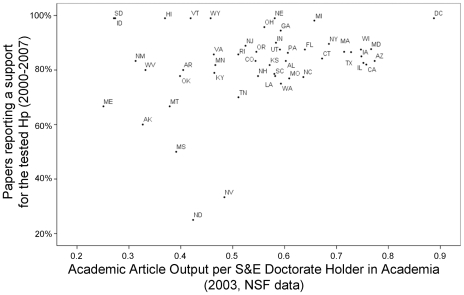
“Positive” results by per-capita publication rate. Percentage of papers supporting a tested hypothesis in each US state plotted against the state's academic article output per science and engineering doctorate holder in academia in 2003 (NSF data). Papers were published between 2000 and 2007 and classified by the US state of the corresponding author. US states are indicated by official USPS abbreviations. For abbreviations legend, see Figure 1.

**Figure 3:**
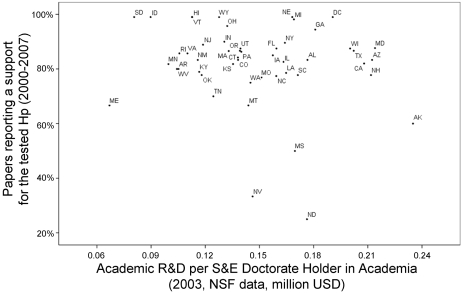
“Positive” results by per-capita R&D expenditure in academia. Percentage of papers supporting a tested hypothesis in each US state plotted against the state's academic R&D expenditure per science and engineering doctorate holder in academia in 2003 (NSF data, in million USD). Papers were published between 2000 and 2007 and classified by the US state of the corresponding author. US states are indicated by official USPS abbreviations. For abbreviations legend, see Figure 1.

The probability of papers to support the tested hypothesis increased significantly with the per capita academic productivity of the state of the corresponding author (b = 1.383±0.682, Wald test = 4.108, df = 1, p = 0.043, Odds-Ratio (95%CI) = 3.988(1.047–15.193), Figure 2). The statistical significance of per capita academic productivity increased when controlling for the per capita R&D expenditure, which tended to have a negative effect instead (respectively, b = 2.644±0.948, Wald = 7.779, p = 0.005, OR(95%CI) = 14.073(2.195–90.241), and b = −5.993±3.185, Wald = 3.539, p = 0.06, OR(95%CI) = 0.002(0–1.285), see Figure 3).

The effect of per capita academic productivity remained highly significant when controlling for expenditure and for characteristics of study: broad methodological category, papers testing one vs. multiple hypotheses, and pure vs. applied discipline (Table 1, Nagelkerke R^2^ = 0.051). Similar results were obtained when controlling for the effect of discipline instead of methodology (Table 2, Nagelkerke R^2^ = 0.065). Adding an interaction term of discipline by academic productivity did not improve the model significantly overall (Wald = 20.424, df = 19, p = 0.369), although contrasting each discipline's interaction term with that of Space Science showed significantly positive interaction effects for Neuroscience & Behaviour (b = 8.098±4.122, Wald = 3.860, p = 0.049) and Pharmacology and Toxicology (b = 11.201±4.661, Wald = 5.775, p = 0.016).

**Table 1:** Logistic regression slope, standard error, Wald test with statistical significance, odds ratio and 95% confidence interval of the probability for a paper to report a positive result, depending on the following study characteristics: per capita academic productivity of US state of corresponding author, per capita R&D academic expenditure of US state of corresponding author, papers testing more than one hypothesis (only the first of which was considered in this study), papers published in pure as opposed to applied disciplines, and methodological category of paper.

Predictor	B	SE	Wald	df	Sig.	OR	95%CI OR
Papers per capita	2.586	0.961	7.235	1	0.007	13.275	2.017–87.368
R&D per capita	−5.603	3.248	2.977	1	0.084	0.004	0–2.142
Multiple hypotheses	−0.839	0.318	6.932	1	0.008	0.432	0.232–0.807
Pure-applied discipline	0.314	0.185	2.886	1	0.089	1.368	0.953–1.965
Methodological category (all)			25.002	4	<0.001		
Biological, Ph/Ch	0.872	0.226	14.850	1	<0.001	2.393	1.535–3.729
Beh/Soc+mixed, non-human	0.465	0.330	1.981	1	0.159	1.592	0.833–3.040
Beh/Soc+mixed, human	1.154	0.285	16.457	1	<0.001	3.172	1.816–5.539
Other methodology	0.080	0.360	0.050	1	0.823	1.084	0.535–2.196
Constant	0.244	0.492	0.245	1	0.621	1.276	

Methodological category (see methods for details) was tested for overall effect, then each category was contrasted by indicator contrast to physical/chemical studies on non-biological material.

**Table 2:** Logistic regression slope, standard error, Wald test with statistical significance, odds ratio and 95% confidence interval of the probability for a paper to report a positive result, depending on the following study characteristics: per capita academic productivity of US state of corresponding author, per capita R&D academic expenditure of US state of corresponding author, papers testing more than one hypothesis (only the first of which was included in the study), and discipline of journal in which the paper was published (as classified by the Essential Science Indicators database, see methods).

Variable	B	SE	Wald	df	Sig.	OR	95%CI OR
Papers per capita	2.509	0.977	6.590	1	0.010	12.292	1.810–83.479
R&D per capita	−5.237	3.263	2.576	1	0.109	0.005	0–3.185
Multiple hypotheses	−0.532	0.344	2.399	1	0.121	0.587	0.299–1.152
Discipline (all)			38.752	19	0.005		
Geosciences	−0.050	0.426	0.014	1	0.906	0.951	0.413–2.192
Environment/Ecology	0.208	0.441	0.223	1	0.637	1.231	0.519–2.920
Plant and Animal Sciences	0.786	0.434	3.284	1	0.070	2.195	0.938–5.135
Computer Science	0.487	0.565	.743	1	0.389	1.627	0.538–4.923
Agricultural Sciences	0.387	0.502	0.596	1	0.440	1.473	0.551–3.939
Physics	0.911	0.577	2.497	1	0.114	2.487	0.803–7.702
Neuroscience & Behaviour	1.139	0.462	6.067	1	0.014	3.124	1.262–7.734
Microbiology	1.163	0.453	6.586	1	0.010	3.198	1.316–7.772
Chemistry	0.781	0.520	2.252	1	0.133	2.183	0.787–6.052
Social Sciences, General	0.917	0.430	4.549	1	0.033	2.503	1.077–5.814
Immunology	1.079	0.463	5.439	1	0.020	2.941	1.188–7.282
Engineering	1.153	0.573	4.048	1	0.044	3.166	1.030–9.731
Mol. Biology & Genetics	0.684	0.447	2.346	1	0.126	1.982	0.826–4.757
Economics & Business	0.952	0.487	3.825	1	0.05	2.591	0.998–6.729
Biology & Biochemistry	0.956	0.481	3.948	1	0.047	2.602	1.013–6.683
Clinical Medicine	1.586	0.531	8.937	1	0.003	4.885	1.727–13.819
Pharm. & Toxicology	1.581	0.508	9.680	1	0.002	4.859	1.795–13.152
Materials Science	1.581	0.565	7.825	1	0.005	4.861	1.605–14.720
Psychiatry/Psychology	1.699	0.563	9.095	1	0.003	5.468	1.813–16.497
Constant	0.147	0.583	0.064	1	0.801	1.159	

Disciplines were tested for overall effect, then each was contrasted by indicator contrast to Space Science.

The proportion of papers published between 2000 and 2007 that supported the tested hypothesis was completely uncorrelated with the total (i.e. non per capita) number of doctorate holders, total number of papers and total R&D expenditure (b = 0±0 and p≥0.223 for all three cases). Controlling for any of these parameters did not alter the results of the regression in any meaningful way.

### Sensitivity analyses

The analyses were run using 2003 data from the Science and Engineering Indicators 2006 report [37], because this year had the most complete data series (all parameters in the report had been calculated for that year), and because it fell almost in the middle of the period 2000–2007. However, state data was also available from the 2004 and 2008 reports, and for the years 2000–2001 and 2005–2006 (year depeding on parameter). Some discrepancies between reports were noted in the data on some states and years (in particular, but not exclusively, for DC). However, similar results were obtained using different data sets or combinations of them. For example, the state productivity averaged over the 2000–2001 and 2005–2006 data series and excluding the 2003 series was still a statistically significant predictor, controlling for expenditure (Per capita number of papers: b = 2.496±1.100, Wald = 5.145, p = 0.023; per capita R&D: b = −6.628±3.742, Wald = 3.138, p = 0.076).

## Discussion

In a random sample of 1316 papers that declared to have “tested a hypothesis” in all disciplines, outcomes could be significantly predicted by knowing the addresses of the corresponding authors: those based in US states where researchers publish more papers per capita were significantly more likely to report positive results, independently of their discipline, methodology and research expenditure. The probability for a study to yield a support for the tested hypothesis depends on several research-specific factors, primarily on whether the hypothesis tested is actually true and how much statistical power is available to reject the null hypothesis [38]. However, the geographical origin of the corresponding author should not, in theory, be relevant, nor should parameters measuring the sheer quantity of publications per capita. Although, as discussed below, not all confounding factors in the study could be controlled for, these results support the hypothesis that competitive academic environments increase not only the productivity of researchers, but also their bias against “negative” results.

All main sources of sampling and methodological bias in this study were controlled for. The number of papers from each state in the sample was almost perfectly correlated with the actual number of papers that each state produced in any given year, which confirms that the sampling of papers was completely randomised with respect to address (as well as any other study characteristic including the particular hypothesis tested and the methods employed), and therefore that the sample was highly representative of the US research panorama. The total number of papers, total R&D and total number of doctorate holders were completely uncorrelated to the proportion of positive results, ruling out the possibility that different frequencies of positive results between states are due to sampling effects. Although the analyses were all conducted by one author, expectancy biases can be excluded, because the classification of papers in positive and negative was completely blind to the corresponding address in the paper, and the US states' data were obtained by an independent source (NSF). We can also exclude that the association between productivity and positive results was an artifact of the effects of methodologies and disciplines of papers (which are elsewhere shown to be significant predictors of positive results [36]), because controlling for these factors increased the size and statistical significance of the regression, suggesting that the effect is truly cross-disciplinary. In sum, these results are likely to represent a genuine pattern characterising academic research in the US.

An unavoidable confounding factor in this study is the quality and prestige of academic institutions, which is intrinsically linked to the productivity of their resident researchers. Indeed, official rankings of universities often include parameters measuring publication rates [39] (although the validity of such rankings is controversial [40], [41]). Therefore, it could be argued that the more productive states are also the ones hosting the “best” universities, which provide better academic structures (laboratories, libraries, etc…) and more advanced and stimulating intellectual environments. This could make scientists better at picking up the right hypotheses and more successful in testing them, increasing their chances to obtain true positive results. Separating this quality-of-institution effect from that of bias induced by pressures to publish is difficult, because the two factors are strictly linked: the best universities are also the most competitive, and thus presumably the ones where pressures to produce are highest.

However, the quality-of-institution effect is unlikely to fully explain the findings of this study for at least two reasons. First, because if structures and resources are really important, then positive results should also tend to increase where more R&D expenditure is available, but a negative (though non statistically significant) trend was observed instead. Second, because the variability in frequency of positive results between states is too high to be reasonably explained by the quality factor alone. At one extreme, states yielded as few as 1 in 4 papers that supported the tested hypothesis, at the other extreme, numerous states reported between 95% and 100% positive results, including academically productive ones like Michigan (N = 54 papers in this sample), Ohio (N = 47), District of Columbia (N = 18) and Nebraska (N = 13). In absence of bias of any kind, this would mean that corresponding authors in these states almost never failed to find a support for the hypotheses they tested. But negative results are virtually inevitable, unless all the hypotheses tested were true, experiments were designed and conducted perfectly, and the statistical power available were always 100% – which it rarely is, and is usually much lower [42], [43], [44], [45], [46].

As a matter of fact, the prestige of institutions could be expected to have the opposite influence on published results, in analogy with what has been observed by comparing countries. In the biomedical literature, the statistical significance of results tends to be lower in papers from high-income countries, which suggests that journal editors tend to reject papers from low-income countries unless they have particularly “good” results [47]. If there were a similar editorial bias favouring highly prestigious universities in the US – and some studies suggest that there is [9], [48] – then the more productive states (prestigious institutions) should be allowed to publish more negative results.

A possibility that needs to be considered in all regression analyses is whether the cause-effect relationship could be reversed: could some states be more productive precisely because their researchers tend to do many cheap and non-explorative studies (i.e. many simple experiments that test relatively trivial hypotheses)? This appears unlikely, because it would contradict the observation that the most productive institutions are also the more prestigious, and therefore the ones where the most important research tends to be done.

What happened to the missing negative results? As explained in the Introduction, presumably they either went completely unpublished or were somehow turned into positive through selective reporting, post-hoc re-interpretation, and alteration of methods, analyses and data. The relative frequency of these behaviours remains to be established, but the simple non-publication of results is unlikely to be the only explanation. If it were, then we should have to assume that authors in the more productive states are even more productive than they appear, but wastefully do not publish many negative results they get.

Since positive results in this study are estimated using what is declared in the papers, we cannot exclude the possibility that authors in more productive states simply tend to write the sentence “test the hypothesis” more often when they get positive results. However, it would be problematic to explain why this should be the case and, if it were, then we would still have to understand if and how negative results are published. Ultimately, such an association of word usage with socio-economic parameters would still suggest that publication pressures have some measurable effect on how research is conducted and/or presented.

Selective reporting, reinterpreting and altering results are commonly considered “questionable research practices”: behaviours that might or might not represent falsification of results, depending on whether they express an intention to deceive. There is no doubt that negative results produced by a methodological flaw should either be corrected or not be published at all, and it is likely that many scientists select or manipulate their negative results because they sincerely think their experiments went wrong somewhere – maybe the sample was too small or too heterogeneous, some measurements were inaccurate and should be discarded, the hypothesis should be reformulated, etc… However, in most circumstances this might be nothing more than a “gut feeling” [49]. Moreover, positive results should be treated with the same scrutiny and rigour applied to negative ones, but with all likelihood they are not. This latter form of neglect is probably one of the main sources of bias in science.

Adding an interaction term of discipline by productivity did not increase the accuracy of the model significantly. Although we are currently unable to measure the statistical power of interaction terms in complex logistic regression models, the lack of significance suggests that large disciplinary differences in the effect of publication pressures are unlikely. Interestingly, however, some interdisciplinary variability was observed: Pharmacology and Toxicology, and Neuroscience and Behaviour had a significantly stronger association between productivity and positive results compared to Space Science. Of course, since we had 20 disciplines in the model, the significance of these two terms could be due to chance alone. However, we cannot exclude that a study with higher statistical power could confirm this result and reveal other small, but nonetheless interesting differences between fields.

This study focused on the United States primarily because they are one of the most scientifically productive countries, and are academically diversified but linguistically and culturally rather homogeneous, which eliminated the confounding effect of editorial biases against particular countries, cultures or languages. Moreover, the research output and expenditure of all US states are recorded and reported by NSF periodically and with great accuracy, yielding a reliable dataset. Academic competition might be particularly high in US universities [1], but is surely not unique to them. Therefore, the detrimental effects of the publish-or-perish culture could be manifest in other countries around the world.

## Materials and Methods

The sample of papers used in this study was part of a larger sample used to compare bias between disciplines [36]. Papers within this latter were obtained with the following method. The sentence “test* the hypothes*” was used to search all 10837 journals in the Essential Science Indicators database, which classifies journals univocally in 22 disciplines. Only papers published between 2000 and 2007 were sampled. When the number of papers retrieved from one discipline exceeded 150, papers were selected using a random number generator. In one discipline, Plant and Animal Sciences, an additional 50 papers were analysed, in order to increase the statistical power of comparisons involving behavioural studies on non-humans (see below for details on methodological categories). By examining the abstract and/or full-text, it was determined whether the authors of each paper had concluded to have found a positive (full or partial) or negative (null or negative) support. If more than one hypothesis was being tested, only the first one to appear in the text was considered. We excluded meeting abstracts and papers that either did not test a hypothesis or for which sufficient information to determine the outcome was lacking.

All data was extracted by the author. An untrained assistant who was given basic written instructions (similar to the paragraph above, plus a few explanatory examples) scored papers the same way as the author in 18 out of 20 cases, and picked up exactly the same sentences for hypothesis and conclusions in all but three cases. The discrepancies were easily explained, showing that the procedure is objective and replicable.

To identify methodological categories, the outcome of each paper was classified according to a set of binary variables: 1-outcome measured on biological material; 2- outcome measured on human material; 3-outcome exclusively behavioural (measures of behaviours and interactions between individuals, which in studies on people included surveys, interviews and social and economic data); 4-outcome exclusively non-behavioural (physical, chemical and other measurable parameters including weight, height, death, presence/absence, number of individuals, etc…). Biological studies in vitro for which the human/non-human classification was uncertain were classified as non-human. Different combinations of these variables identified mutually exclusive methodological categories: Physical/Chemical (1-N, 2-N, 3-N, 4-Y); Biological, Non-Behavioural (1-Y, 2-Y/N, 3-N, 4-Y); Behavioural/Social (1-Y, 2-Y/N, 3-Y, 4-N), Behavioural/Social + Biological, Non-Behavioural (1-Y, 2-Y/N, 3-Y, 4-Y), Other methodology (1-Y/N, 2-Y/N, 3-N, 4-N). Disciplines were attributed based on how the ESI database had classified the journal in which the paper appeared, and the pure-applied status of discipline followed classifications identified in previous studies (for further details see [36]).

From this larger sample, all papers with a corresponding address in the US were selected, and the US state of each was recorded. Data on state academic R&D expenditure, number of doctorate holders in academia and number of papers published were taken directly from the State Indicators section of the Science and Engineering Indicators 2006 report [37]. This report compiles data from three different sources: Thomson ISI - Science Citation Index and Social Sciences Citation Index; National Science Foundation, Division of Science Resources Statistics - Survey of Doctorate Recipients; National Science Foundation, Division of Science Resources Statistics - Academic Research and Development Expenditures. When counting the number of papers by state, NSF corrects for multiple authorship by dividing each paper by the number of institutions involved. The scoring of papers as “positive” and “negative” was completely blind to the corresponding author's address. As explained in the Results section, data from other reports were extracted and used for sensitivity analyses.

### Statistical analyses

The ability of independent variables to predict the outcome of a paper was tested by standard logistic regression analysis, fitting a model in the form:
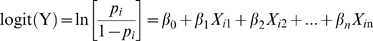
in which *p_i_* is the probability of the *i*th paper of reporting a positive result, X_1_ is the number of papers published per capita (per doctorate holder in academia) in the state of the corresponding author of the *i*th paper, X_2_ is the *i*th paper's state R&D expenditure per capita, and X_n_ represents the various characteristics of the *i*th paper that were controlled for in the models (e.g. dummy variables for methodology, discipline, etc…) as specified in the Results section. Statistical significance of the effect of each variable was calculated through Wald's test. Except where specified, all parameter estimates are reported with their standard error. The relative fit of regression models was estimated with Nagelkerke's adjusted R^2^.

Multicollinearity among independent variables was tested by examining tolerance and Variance Inflation Factors for all variables in the model. All variables had tolerance≥0.42 and VIF≤2.383 except one of the methodological dummy variables (Tolerance = 0.34 and VIF = 2.942). To avoid this (modest) sign of possible collinearity, methodological categories were reduced to the minimum number that previous analyses have shown to differ significantly in the frequency of positive results: purely physical and chemical, biological non-behavioural, and behavioural and mixed studies on humans and on non-humans [36]. This removed any presence of collinearity in the model. All analyses were produced using SPSS statistical package.

### Figures

Confidence intervals in the graphs were obtained independently from the statistical analyses, using the following logit transformation to calculate the proportion of positive results and standard error:
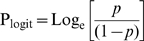


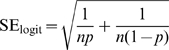
Where *p* is the proportion of negative results, and *n* is the total number of papers. Values for high and low confidence interval were calculated and the final result was back-transformed in percentages using the following equations for proportion and percentages, respectively:
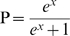


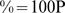
Where *x* is either P_logit_ or each of the corresponding 95%CI values.
